# Ultrasonography and Atypical Sites of Endometriosis

**DOI:** 10.3390/diagnostics10060345

**Published:** 2020-05-27

**Authors:** Stefano Guerriero, Francesca Conway, Maria Angela Pascual, Betlem Graupera, Silvia Ajossa, Manuela Neri, Eleonora Musa, Marcelo Pedrassani, Juan Luis Alcazar

**Affiliations:** 1 Obstetrics and Gynecology, University of Cagliari, 09124 Cagliari, CA, Italy; francesca.conway@gmail.com (F.C.); gineca.sajossa@tiscali.it (S.A.); manu.neri11@hotmail.it (M.N.); emusa87@gmail.com (E.M.); 2 Department of Obstetrics and Gynecology, Azienda Ospedaliero Universitaria, Policlinico Universitario Duilio Casula, 09045 Monserrato, CA, Italy; 3 Department of Obstetrics, Gynecology and Reproduction, Hospital UniversitariDexeus, 08028 Barcelona, Spain; marpas@dexeus.com (M.A.P.); BETGRA@dexeus.com (B.G.); 4 Hospital Maternidade Carmela Dutra and ClinusClínica de Imagem, Florianopolis 88015–270, Brazil; marcelo.pedrassani@gmail.com; 5 Department of Obstetrics and Gynecology, Clínica Universidad de Navarra, School of Medicine, University of Navarra, 31008 Pamplona, Navara, Spain; jlalcazar@unav.es

**Keywords:** scar endometriosis, endometriosis of the rectus muscle, inguinal endometriosis, perineal endometriosis, appendiceal endometriosis, hepatic endometriosis, endometriosis of pancreas, endometriosis of kidney, diaphragmatic endometriosis, peripheral nerves endometriosis

## Abstract

In the present pictorial we show the ultrasonographic appearances of endometriosis in atypical sites. Scar endometriosis may present as a hypoechoic solid nodule with hyperechoic spots while umbilical endometriosis may appear as solid or partially cystic areas with ill-defined margins. In the case of endometriosis of the rectus muscle, ultrasonography usually demonstrates a heterogeneous hypoechogenic formation with indistinct edges. Inguinal endometriosis is quite variable in its ultrasonographic presentation showing a completely solid mass or a mixed solid and cystic mass. The typical ultrasonographic finding associated with perineal endometriosis is the presence of a solid lesion near to the episiotomy scar. Under ultrasonography, appendiceal endometriosis is characterized by a solid lesion in the wall of the small bowel, usually well defined. Superficial hepatic endometriosis is characterized by a small hypoechoic lesion interrupting the hepatic capsula, usually hyperechoic. Ultrasound endometriosis of the pancreas is characterized by a small hypoechoic lesion while endometriosis of the kidney is characterized by a hyperechoic small nodule. Diaphragmatic endometriosis showed typically small hypoechoic lesions. Only peripheral nerves can be investigated using ultrasound, with a typical solid appearance. In conclusion, ultrasonography seems to have a fundamental role in the majority of endometriosis cases in “atypical” sites, in all the cases where “typical” clinical findings are present.

## 1. Introduction 

Most commonly, endometriosis affects the ovaries, pelvic peritoneum, uterosacral ligaments, fallopian tubes, and broad ligaments [1]. Unfortunately, extragenital implants of endometriosis can be spotted virtually in any other pelvic compartment [2]; in fact, extra-pelvic foci of ectopic endometrial tissue have been described in almost every organ and tissue of the body [3].

Extra-pelvic endometriosis remains an unclear clinical entity with an unknown prevalence, due to the absence of rigorously conducted epidemiological studies [4] and a lack of consensus with regards to a gold standard diagnostic technique. Endometriosis is characterized by a highly variable clinical presentation given the multiple areas that can be involved. Hence, clinicians should be aware of the fact that any symptom affecting extra-pelvic sites and described by a patient of child-bearing age as “cyclical” might be a possible indicator of endometriosis and deserves further investigation.

The main typical sites include extra-pelvic and extragenital endometriosis and this can be classified as suggested by Andres et al. [5]:

### 1.1. Abdominal

1. Parietal endometriosis (PE) included primary lesions that involve the abdominal wall (scar endometriosis, Villar’s nodule, rectus abdominis endometriosis), groin (canal of Nuck endometriosis) and perineum.

2. Visceral endometriosis (VE): bowel, liver, pancreas, kidney, and gallbladder. While the rectosigmoid colon is the most common location (52–72%) but has to be included in pelvic endometriosis, endometriotic implants can also be found in the small bowel, especially in the terminal ileum (4.1–16.9%). As a matter of fact, the most common location of extra-pelvic intestinal endometriosis is the last part of the ileum (the small intestine), the cecum (the first part of the large bowel), and the appendix [6].

### 1.2. Thoracic

Thoracic endometriosis (TE) is an endometriosis lesion that involves the diaphragm, pleura, and lung.

### 1.3. Other Locations

There are other (non-abdominal and non-thoracic) sites, such as vascular, lymphatic, and central nervous systems, which are localizations of endometriosis, but the majority of these sites are virtually impossible to evaluate using ultrasound.

The majority of studies have been performed using MRI as reported in the systematic review of Andres et al. [5] and few studies have showed a wide range of ultrasound images [7,8] with a possible variety of findings. The aim of the present study is to perform a pictorial ultrasound essay of endometriosis in atypical sites. In fact, the identification of endometriosis in extragenital locations has to be performed by the gynecologist and radiologist using a less expensive approach than MRI. We will show the different ultrasonographic findings associated with this disease in these sites.

## 2. Abdominal Endometriosis

### 2.1. Parietal Endometriosis (PE) or Abdominal Wall Endometriosis

Occasionally, ectopic endometrial tissue can be found within the abdominal wall structures. This finding is often associated with a history of previous laparoscopic or laparotomic hysterotomy. This condition is mainly iatrogenic and is related to the uterine cavity incision closure. To prevent this occurrence, for example during a C-section, the surgeon should avoid including the endometrium within the suture when closing the layer of the myometrium.

Nonetheless, not all cases of abdominal wall endometriosis are related to surgery. Although it is true that patients with abdominal wall endometriosis have a high incidence of previous C-sections, it may be equally possible that the endometrium may become particularly susceptible to mechanisms such as transplantation and implantation during pregnancy, promoting the development of endometriosis [2].

Abdominal wall endometriosis can present as a painful and tender mass, with a tendency to grow in size, characterized by increasing pain and possible bleeding during menses [9] and might be confused with conditions such as a suture granuloma, an incisional hernia or a primary or metastatic cancer [9]. Medical therapy offers a temporary solution since patients often report a recrudescence of symptoms after suspending the drug. Surgical excision is therefore recommended for abdominal wall endometriosis [9].

### 2.2. Scar Endometriosis

**Figure 1:**
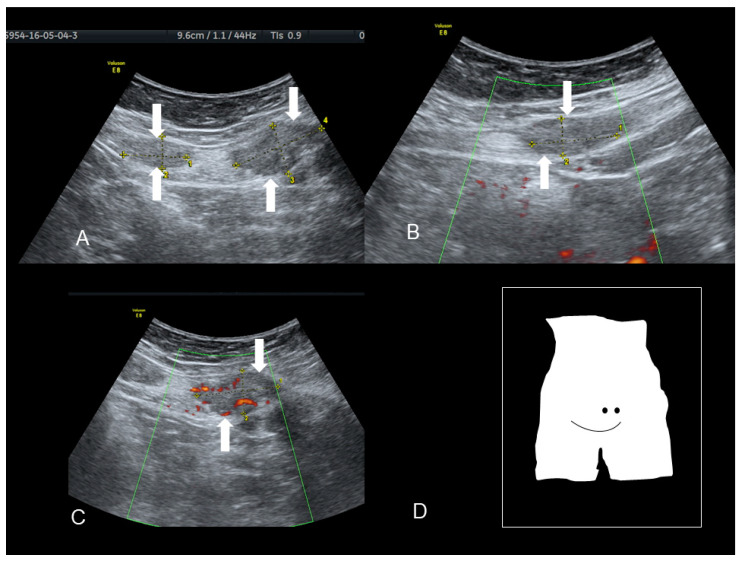
Ultrasonographic images of two nodules (**A**–**C**) (see white arrows), of scar endometriosis with some color spots (C) due to internal vascularization, infiltrating the external oblique muscle in a woman with a previous cesarean section some years before. Drawing of the location (**D**).

**Figure 2:**
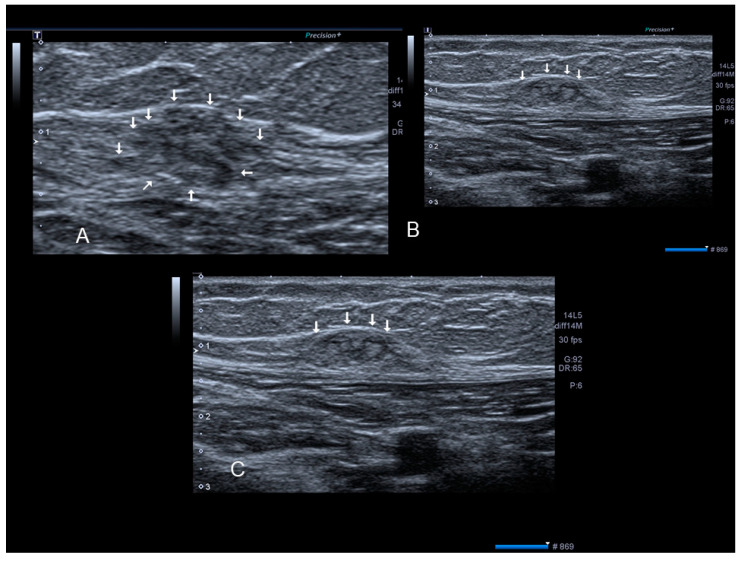
Ultrasonographic images of one nodule (**A**–**C**) (see thin with arrows), of scar endometriosis infiltrating the external oblique muscle in a woman with a previous cesarean section some years before cesarean scar endometriosis.

This condition includes all cases of endometrial tissue spread within a surgical wound, with reports of ectopic endometrium found within C-section scars, episiotomy scars following spontaneous delivery, and in surgical scars produced by any surgical intervention that may involve manipulation of the endometrial cavity, such as hysterectomy, salpingotomy for ectopic pregnancies and procedures conducted in the first trimester of pregnancy or during the early second trimester [10]. The ectopic tissue can disseminate within the uterine scar or in the thickness of the abdominal muscles or of the subcutaneous tissue. The reported incidence of scar endometriosis is about 3.5% in patients who undergo gynecological surgery and about 0.8% in all women with a previous C-section [10]. Cesarean sections represent the strongest risk factor for scar endometriosis considering the close contact that may commonly occur between endometrial cells and the subcutaneous during this type of surgery [10]. Nonetheless, the incidence of scar endometriosis following a C-section may be underreported since it is not easily diagnosed. Following surgery, the lesion may take from six months to several years to develop and may not present as a palpable and tender mass. Although an abdominal solid mass spotted at ultrasound cannot be immediately considered as endometriosis, if the lesion is located in close proximity to the C-section scar, endometriosis should be included in the differential diagnosis (Figure 1 and Figure 2) [9].

Scar endometriosis may present as a hypoechoic solid nodule with hyperechoic spots or strands which represent fibrosis within the scar tissue, a hyperechoic peripheral ring, spiculated borders and a single vessel entering the nodule from the periphery (Figure 1 and Figure 2) [11,12,13]. In nodules larger than 30 mm, cystic portions and/or fistulous tract, loss of oval or round shape, multiple vascular pedicles, and central vascularization are more frequent [12].

A malignant transformation of endometriosis represents a very rare occurrence. Malignant transformation has been reported in 1% of endometriosis cases and mainly involves ovarian endometriosis; in fact, 80% of endometriosis-associated malignancies take place in ovaries affected by endometriomas [14]. There have been reports of malignancies occurring in abdominal scar endometriosis, although this transformation is quite rare and accounts for only 4.5% of all extra-genital endometriosis-associated malignancies; in such cases, the most commonly reported histotype is represented by clear cell histology [15]. A recent case series has described 23 cases of clear cell carcinoma arising from cesarean section scars [16]. This malignancy, although rare, appears to be on the rise probably due to a greater knowledge of the disease, as well as an increase in the rate of cesarean sections and uterine surgeries over the past years.

A detailed collection and assessment of patient history are extremely helpful and can guide the physician towards the diagnosis of an endometriosis-associated malignancy. Considering the rarity of this condition and the diagnostic difficulties it is not surprising that endometriosis-associated malignancies tend to reach very large dimensions before a correct diagnosis is formulated [16].

### 2.3. Umbilical Endometriosis or Villar’s Nodule

**Figure 3:**
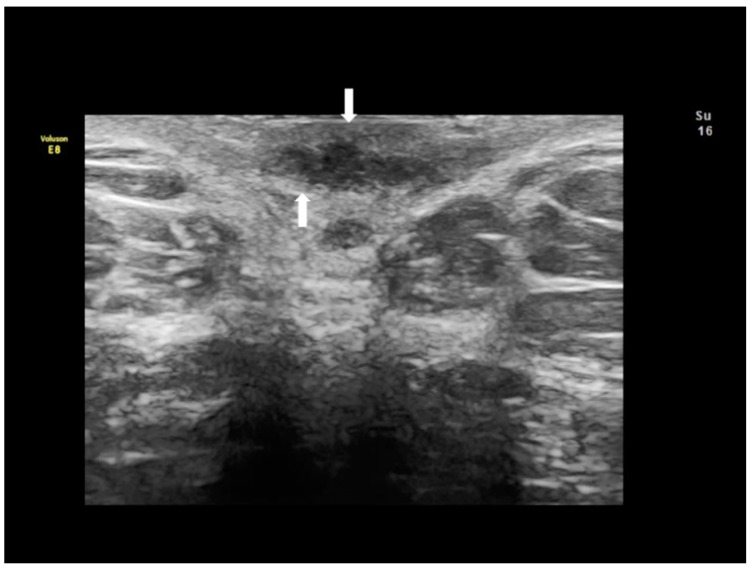
The ultrasonographic appearance of a Villar’s nodule (see white arrows) in a woman without previous abdominal surgery.

**Figure 4:**
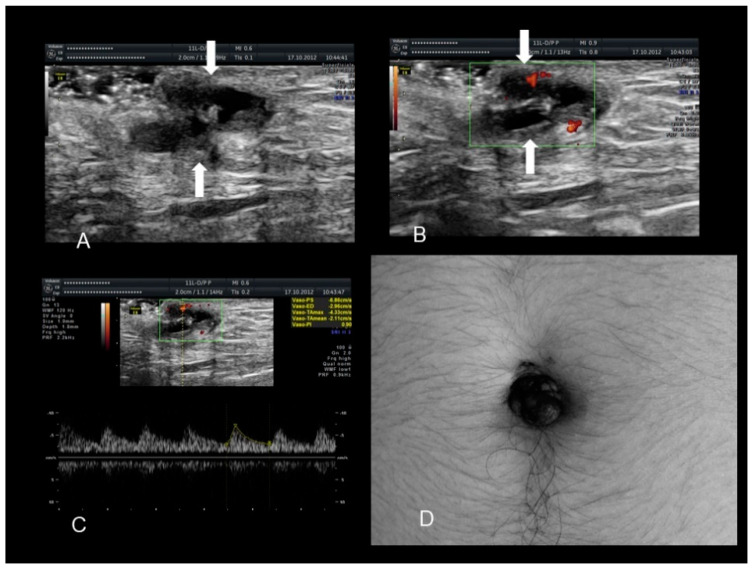
The ultrasonographic appearance of a Villar’s nodule (see white arrows) using B-mode (**A**) and color Doppler (**B**,**C**) with some color spots due to peripheral vascularization in a woman without previous abdominal surgery. In this case the ultrasonographic appearance was more cystic than solid (**A**–**C**). The nodule at visual evaluation (**D**).

**Figure 5:**
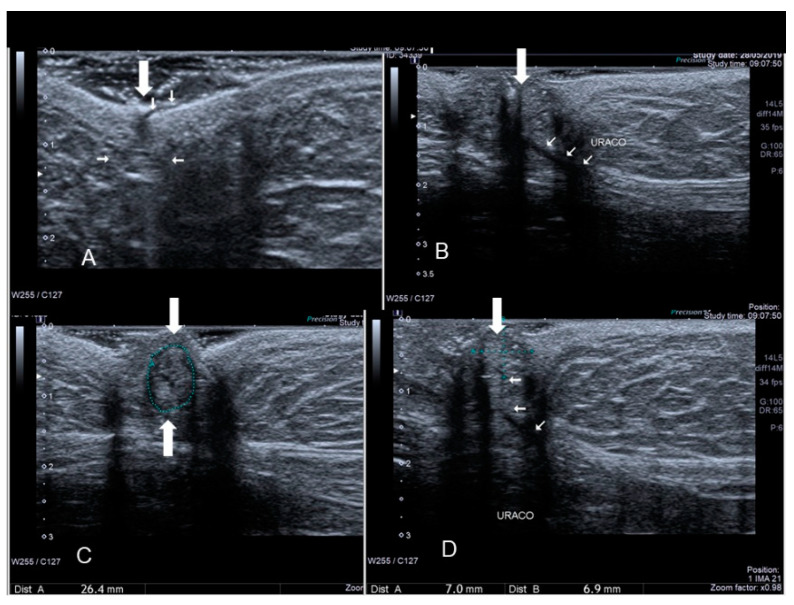
The ultrasonographic appearance of another Villar’s nodule (thick and thin white arrows) (**A**–**D**).

Villar’s nodule is characterized by the presence of endometrial tissue in the umbilicus as a result of tissue seeding during surgery (Figure 3, Figure 4 and Figure 5). It is a rare condition and it is iatrogenic in the vast majority of cases, correlated to previous abdominal/uterine surgery, although there have been very few reports of umbilical endometriosis without a history of previous pelvic surgery (Figure 3) [17].

This site of extra-genital endometriosis was reported for the first time by Villar in 1886 and since then about 100 cases have been described in the literature. It represents about 0.5–1% of all extra-pelvic locations of endometriosis [18]. Umbilical endometriosis usually appears in patients of reproductive age as a solitary lesion, in the absence of concomitant pelvic locations of disease, unlike other types of endometriosis [18]. From a morphological point of view it presents as a small bluish-pink mass, with a diameter ranging from a few millimeters up to 6 cm, a tendency to swell and cause painful symptoms, particularly in the premenstrual period, and possibly bleeding through the skin covering the umbilicus during menses, earning the title of “menstruating tumor”(Figure 3, Figure 4 and Figure 5).

This umbilical endometriosis may appear as solid areas with ill-defined margins (Figure 3, Figure 4 and Figure 5) that can be irregular or spiculated [11], but more frequently than other endometriotic lesions they may have a cystic appearance (although Nuck nodules may also have this appearance) [18] (Figure 3, Figure 4 and Figure 5). In fact, the typical US finding is a nodular formation that occupies the umbilical scar, with ground-glass echogenicity, irregular margins, and no papillary structures with a detectable blood flow [7]. The absence of continuity with the deep fascial plane allows its differential diagnosis from invasive malignancies and hernias [18].

### 2.4. Rectus Abdominis Endometriosis

**Figure 6:**
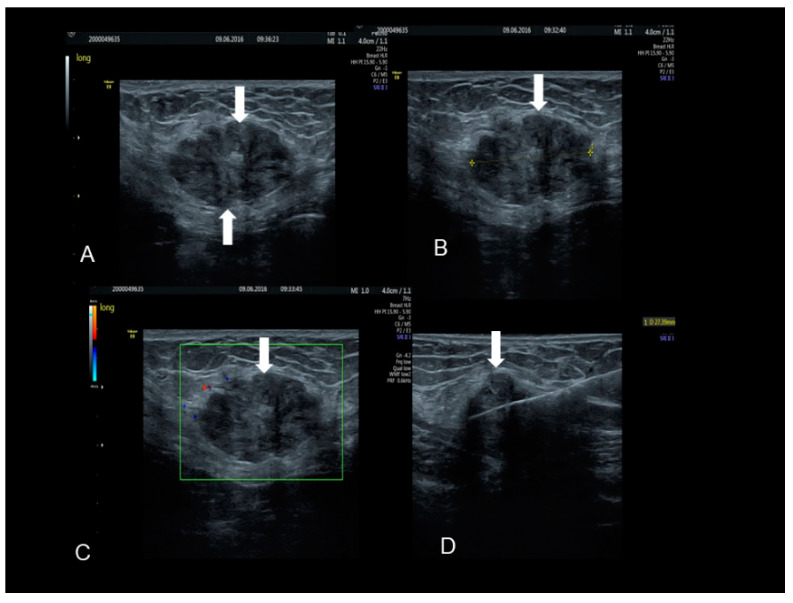
The ultrasonographic appearance of a rectus abdominis endometriotic nodule (see white arrows) (**A**–**D**) in a woman with previous cesarean section and hysterectomy. The last picture (**D**) is taken during the needle biopsy.

**Figure 7:**
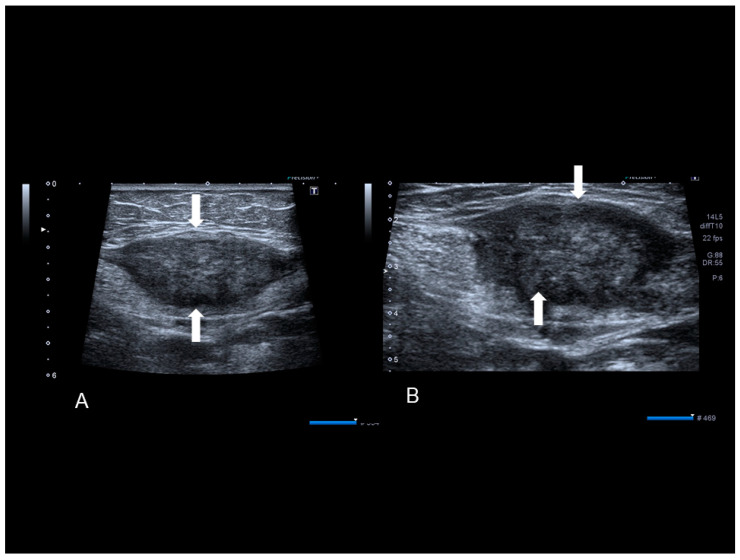
A rectus abdominis endometriosis (white arrows) in a woman without previous surgery (**A**,**B**).

**Figure 8:**
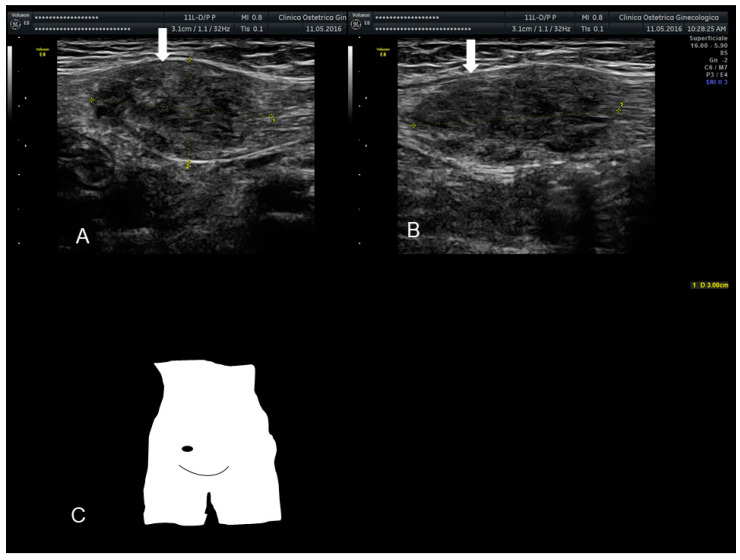
The ultrasonographic appearance of a rectus abdominis endometriosis (see white arrows) (**A**,**B**) in a woman with one previous cesarean section. Drawing of the location (**C**).

Endometriosis of the rectus muscle is an extremely rare localization of extra-pelvic endometriosis [19]. Ultrasonography usually demonstrates a heterogeneous hypoechogenic formation with indistinct edges [20] (Figure 6, Figure 7 and Figure 8). To help in the diagnosis, if the patient moves her legs alternatively during the examination the operator can easily visualize the muscular layers.

### 2.5. Inguinal Endometriosis or Canal of Nuck Endometriosis

**Figure 9:**
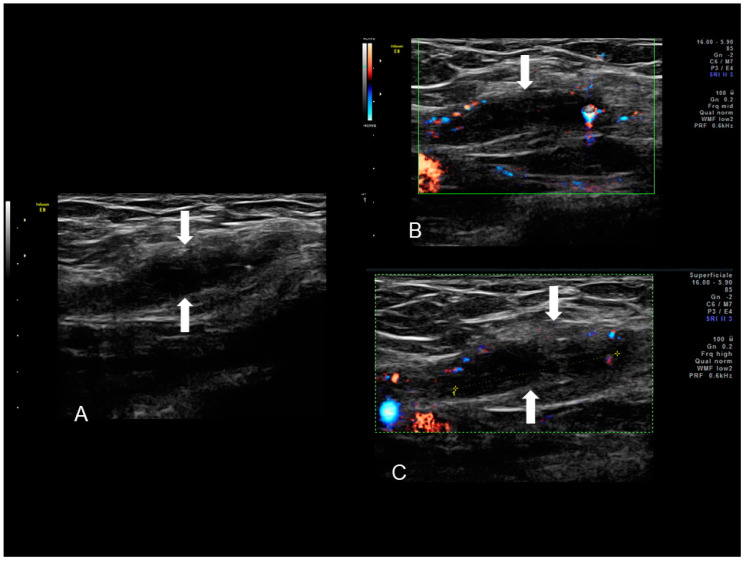
Sonographic features of right inguinal endometriosis (see white arrows) (**A**) presenting as a cystic mass with internal septa, hypoechoic content and few peripheral color spots located in inguinal area due to a scanty vascularization (**B**,**C**).

**Figure 10:**
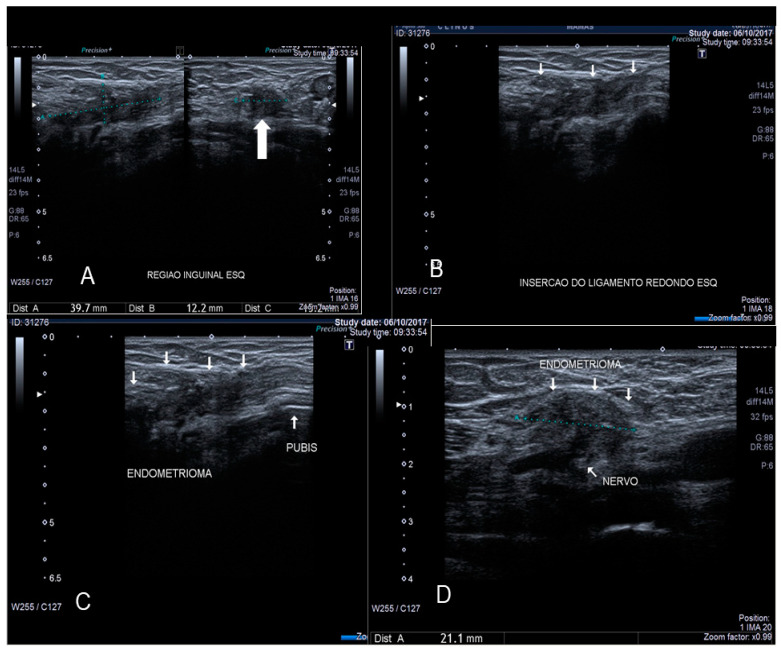
Sonographic features of solid inguinal endometriosis (see thin and thick with arrows) (**A**–**D**).

The canal of Nuck is a small protrusion of the parietal peritoneum which is attached to the uterus by the round ligament through the internal inguinal ring into the inguinal canal. Cullen first reported endometriosis of the inguinal canal in 1896. This site of endometriosis is quite rare, with a reported incidence of about 0.6% [21]. The inguinal canal may become a site of ectopic implants of endometrial tissue by direct extension of the endometrial cells from the uterine cavity to the inguinal canal through the round ligament which may, on occasion, remain patent, thus creating a communication between the peritoneal cavity and the inguinal canal [22] (Figure 9 and Figure 10). Inguinal endometriosis usually occurs on the right side (90% of cases), often alongside an inguinal hernia. It may be suspected in the presence of an inguinal mass that may swell and cause painful symptoms, particularly during menses [21].

Inguinal endometriosis is quite variable in its ultrasonographic presentation. It may appear as a completely solid mass (Figure 9) or a mixed solid and cystic mass (Figure 10). Cystic masses can be hypo or hyperechoic [22] and usually there is evidence of peripheral vascularization at Doppler evaluation (Figure 9).

### 2.6. Perineal Endometriosis

**Figure 11:**
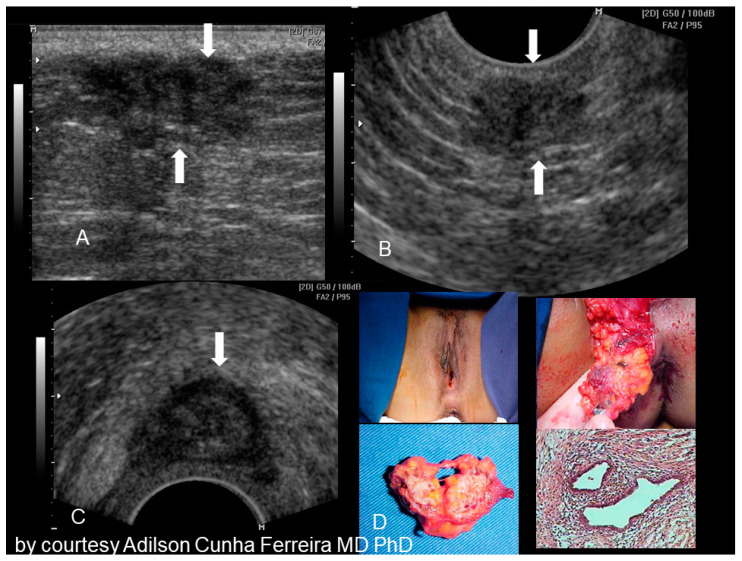
Sonographic features of perineal endometriosis (see white arrow) (**A**–**C**). The visual appearance, the specimen, and the pathology picture (**D**) (courtesy of Adilson Cunha Ferreira, MD, PhD).

Endometriosis of the perineum and vulva is extremely rare, with the most common site being episiotomy scars. Color Doppler ultrasound revealed a subcutaneous nodule with an irregular outline and echo-complex density underlying the episiotomy scar [23]. Episiotomy scar endometriosis represents the less frequent type of scar endometriosis with a much lower prevalence compared to endometriosis in abdominal wall scars [9]. The typical ultrasonographic finding associated with perineal endometriosis is the presence of a solid lesion near to the episiotomy scar (Figure 11).

## 3. Intra-Abdominal or Visceral Endometriosis

Every abdominal organ can be affected by endometriosis. Various case reports have documented endometriosis findings within the appendix, the liver and the diaphragm. In addition, the kidney can be involved. Oddly, the spleen has never been indicated as a site of extra-pelvic endometriosis, despite its relevant immunological role [2].

### 3.1. Endometriosis of the Appendix

**Figure 12:**
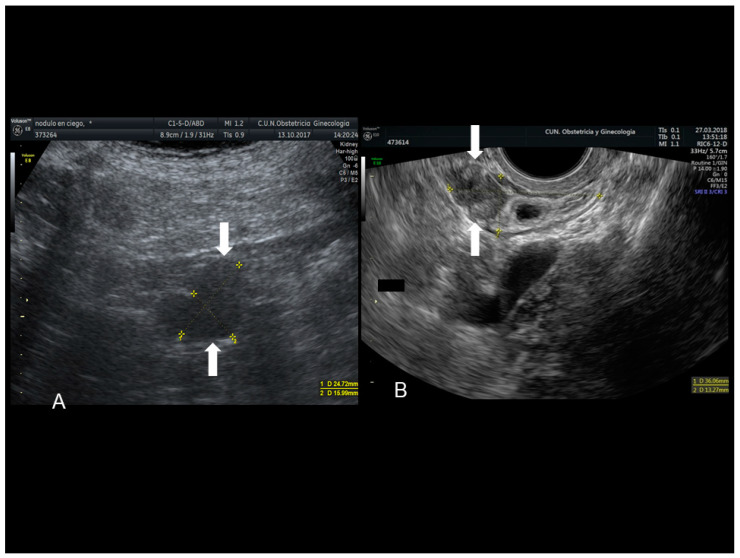
Sonographic features of an endometriotic nodule of cecum (see white arrow) (**A**) and appendix (see white arrow) (**B**).

**Figure 13:**
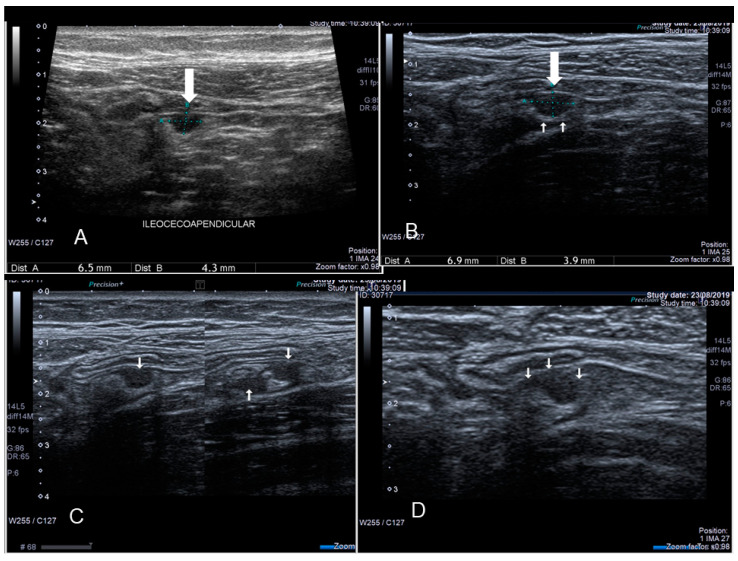
Endometriosis of appendix (see thin and thick white arrow) confirmed at surgery (**A**–**D**).

**Figure 14:**
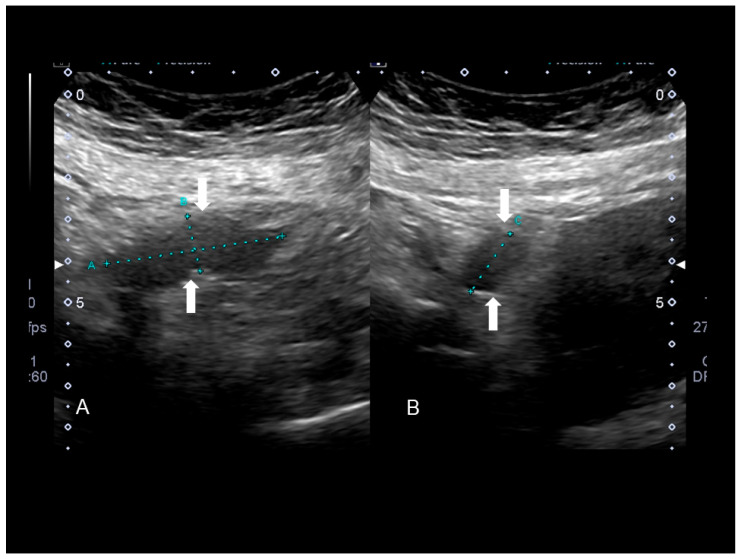
Endometriosis of appendix (see white arrow) confirmed at surgery (**A**,**B**).

**Figure 15:**
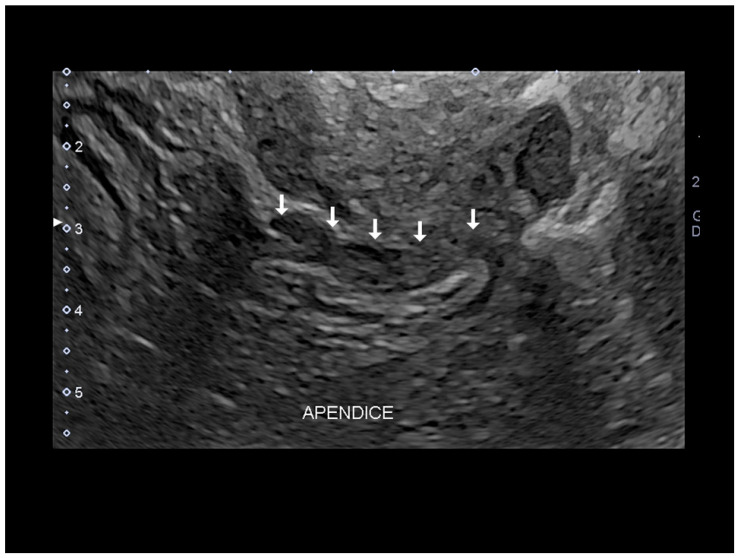
Endometriosis of appendix (see white arrow) by transvaginal endometriosis confirmed at surgery.

The appendix is also an unusual site of extra-pelvic endometriosis. Endometriotic lesions affecting this area may be asymptomatic or may provoke acute appendicitis, melena, ruptured bowel or obstructed bowel from intussusception. Appendiceal endometriosis has a typical histopathological appearance: the mucosa is always left unscathed while the endometrial glands, stroma and hemorrhagic foci are gathered in the muscular and seromuscular layers [24]. Appendiceal endometriosis may have similarities with the clinical presentation of diverticular disease, colorectal cancer, inflammatory bowel disease, carcinoid tumors, benign intramural neoplasms, occult intra-abdominal metastases, mesenteric neoplasms and pelvic abscesses [25]. In ultrasonography appendiceal endometriosis is characterized by a solid lesion in the wall of small bowel, usually well defined (Figure 12, Figure 13, Figure 14 and Figure 15).

### 3.2. Endometriosis of the Small Intestine

**Figure 16:**
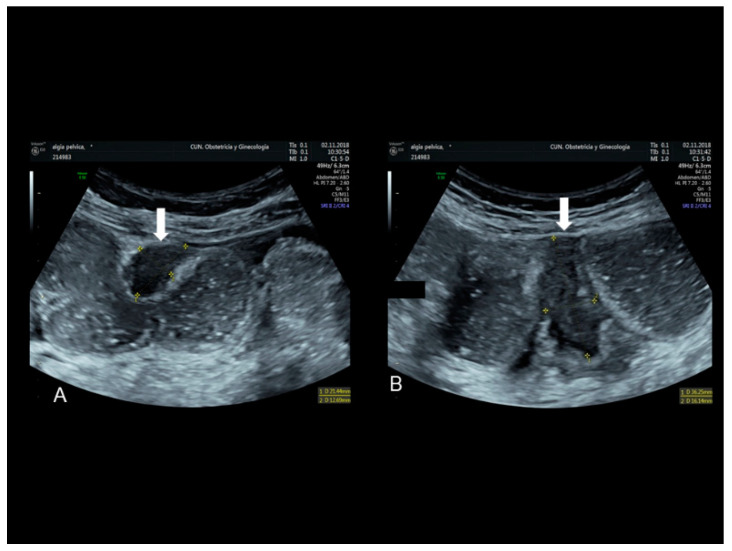
Endometriosis of ileum (see white arrow) confirmed at surgery with bowel dilatation (**A**,**B**).

Endometriosis of the ileum (the small intestine) and the cecum is characterized by the presence of a hypoechoic well-defined lesion located in the cecum (Figure 16) or involving to the ileum [26,27]. There might be multifocal lesions (Figure 16). Clinically, this endometriotic implant may cause intestinal obstruction [28].

### 3.3. Hepatic Endometriosis

**Figure 17:**
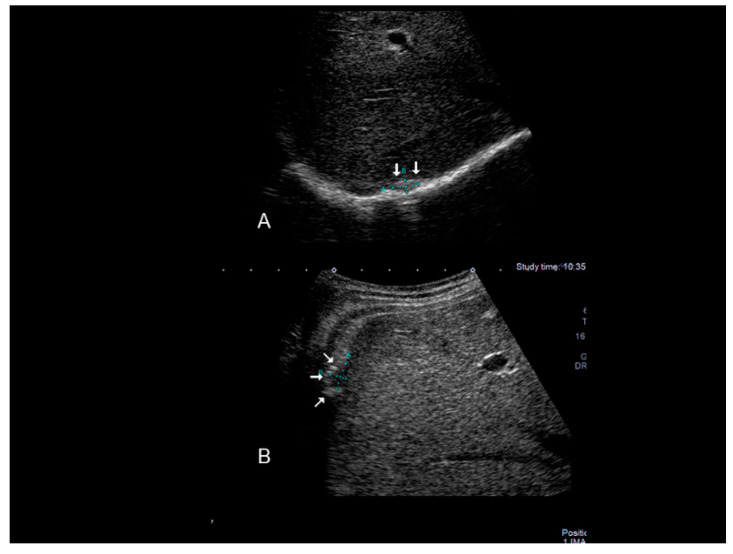
Hepatic superficial endometriosis (see white arrow) confirmed at surgery (**A**,**B**).

This condition is one of the rarest sites of extra-pelvic endometriosis with only 22 reported cases up to now. How the endometrial cells reach the liver is unclear although mechanisms of lymphatic spread of endometrial tissue have been hypothesized. Endometriotic lesions within the hepatic parenchyma can be visualized through ultrasound, computerized tomography (CT) and magnetic resonance imaging (MRI) albeit no pathognomonic features have been described for any of these imaging techniques, therefore a definite diagnosis is completely reliant on histopathological examination [29]. Liver endometriosis is also hard to diagnose and its appearance through imaging techniques may mimic an echinococcal cysts, an abscess, a hematoma, a cystadenoma or a malignancy such as cystadenocarcinoma, or a metastasis [26]. On the contrary superficial hepatic endometriosis is characterized by small hypoechoic lesion interrupting the hepatic capsula, usually hyperechoic (Figure 17).

### 3.4. Endometriosis of Pancreas

**Figure 18:**
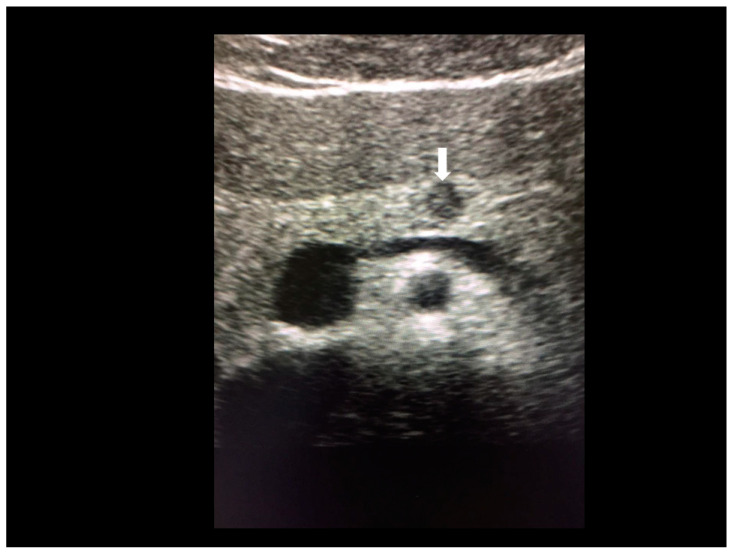
Endometriosis of pancreas (arrow) confirmed at MRI.

Endometriosis of pancreas is associated with epigastric pain or acute pancreatitis [5,30]. In ultrasound, endometriosis of pancreas is characterized by small hypoechoic lesion (Figure 18).

### 3.5. Endometriosis of Kidney

**Figure 19:**
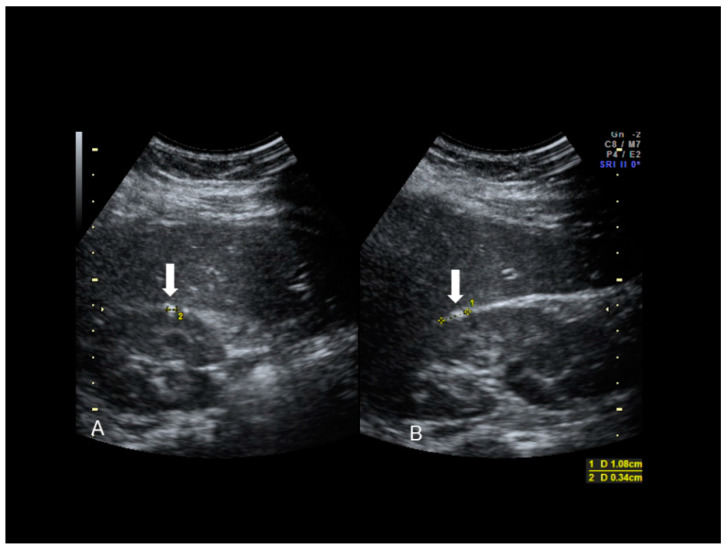
Endometriosis of kidney (see white arrow) confirmed at MRI (**A**,**B**).

Endometriosis of kidney is very rare with only ten cases reported in the literature [5,31]. This kind of endometriosis shows a hyperechoic small nodule (Figure 19).

### 3.6. Thoracic Endometriosis

**Figure 20:**
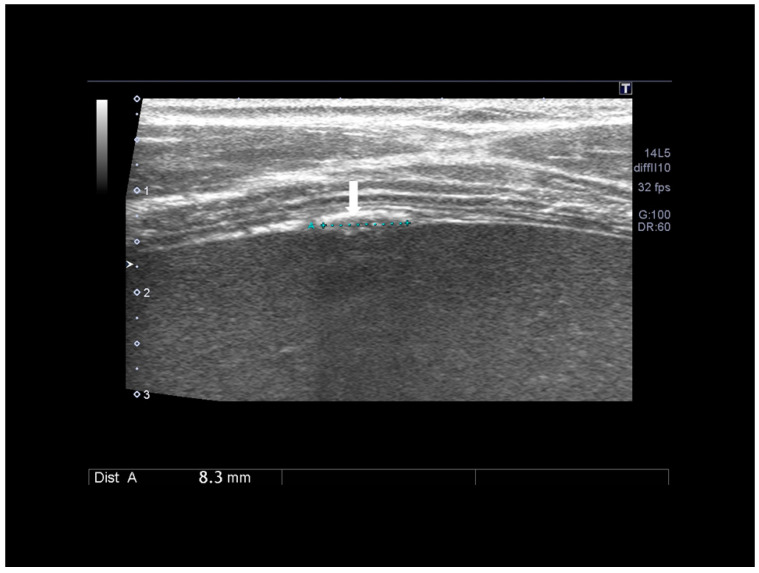
Diaphragmatic small nodule of endometriosis (see white arrow) confirmed at surgery.

**Figure 21:**
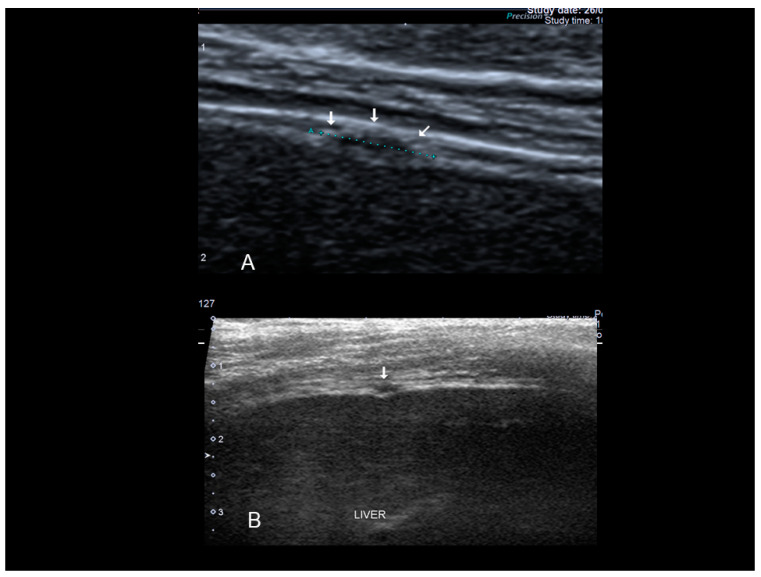
Diaphragmatic endometriosis (see white arrow) confirmed at surgery (**A**,**B**).

Thoracic endometriosis (TE) is an endometriotic lesion that involves the diaphragm, pleura, and/or lung. Lung is rare, but diaphragm lesions are not so rare [5,7,32]. The typical manifestation of diaphragmatic endometriosis is catamenial pneumothorax. Usually the diagnosis is performed using MRI in the absence of reports regarding ultrasound findings. Diaphragmatic endometriosis is characterized by ultrasound as small hypoechoic lesion (Figure 20 and Figure 21).

### 3.7. Other (Nonabdominal and Nonthoracic) Sites: Vascular, Lymphatic, and Central and Peripheal Nervous Systems

**Figure 22:**
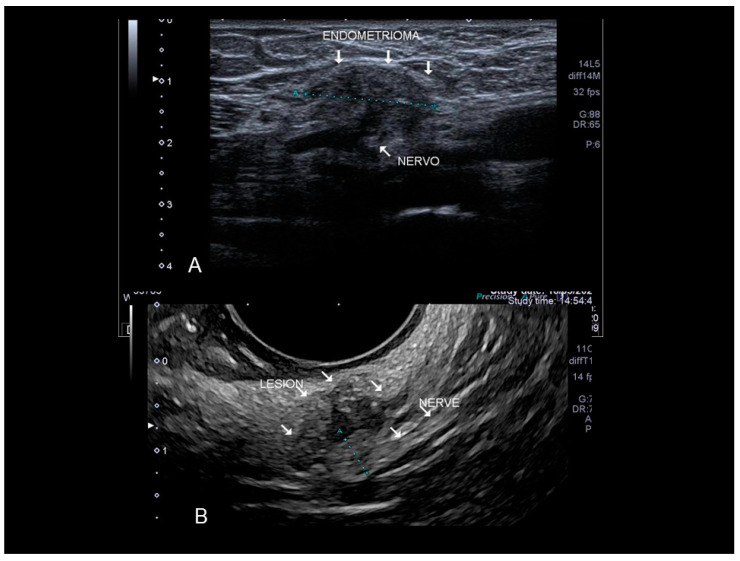
Endometriosis of very lateral retroperitoneal implant (see white arrow) in the topography of Latzko space with nerve involvement confirmed at surgery. The clearest portion measured probably refers to the compromised epineurium. Findings confirmed by MRI and electroneuromyography (**A**). The last picture is a nerve branch involvement by wall endometrioma in the topography of external iliac vessels (see white arrow) (**B**). Confirmed by surgery.

Only peripheral nerves can be investigated using ultrasound [33]. The most investigated nerve, due to the typical symptomatology, is the sciatic nerve. Ultrasonography, extending from the sciatic notch to the level of the ischial tuberosity, shows that the sciatic nerve was “engulfed” in a large, perineural, hypoechogenic, inhomogeneous lesion with an irregular contour corresponding to an endometrioma. Other nerves can be involved. We observed retroperitoneal implant in the topography of Latzko space (also called lateral pararectal space) with nerve involvement (Figure 22).

## 4. How to Avoid Mistakes in the Diagnosis of Endometriosis in Atypical Sites

A careful collection of patient history is a crucial step for the assessment of patients with suspected endometriosis, as well as a thorough evaluation of all the reported symptoms. Patient history should include: family history of endometriosis; previous myomectomy or Cesarean delivery (the most important risk factor for extra-pelvic endometriosis); previous surgery for endometriosis; previous non-surgical therapies for endometriosis (type, duration of treatment and effects); pain should always be investigated (type of pain, localization and occurrence during menses); and finally oral contraceptive assumption should be reported considering it might mask the presence of painful symptoms. Usually, pain levels are described through visual analogue scale (VAS) or through a numerical rating scale (NRS) ranging from 0–10 [34].

In addition, the selection of the correct probe can reduce the risk of misdiagnosis. The best definition of lesion margins and vascularity can be obtained using the highest possible frequency; abdominal wall endometriosis can be spotted using a trans-abdominal approach with a linear transducer (5.0–13.0 MHz). If the depth of the lesion allows, surgical excision should preferably be ultrasound-guided, using a linear superficial probe.

When performing the ultrasound examination the operator should not only characterize the sonographic appearance but also report the number of lesions and their localization (right, left or median quadrant; at the umbilical level; right or left inguinal canal), their depth (superficial lesion contained in the subcutaneous fatty tissue or deep lesion affecting the muscles—in this case the relationship with the fascia should also be specified) and finally the proximity to a previous C-section scar. Lesion diameters should always be measured in the three orthogonal planes. Assessment of lesion vascularity through power Doppler examination with a pulse-repetition frequency of 500–750 MHz is part of the lesion characterization; a moderate/high color score may be suggestive of malignancy.

In addition, the examiner should keep in mind that extra-pelvic endometriosis can be multifocal: always check for multiple sites of the disease. For these reasons, always perform a detailed transvaginal examination using the International Deep Endometriosis Analysis (IDEA) consensus [34] to rule out possible pelvic implants of endometriosis. A transvaginal scan should be conducted in all patients with extra-pelvic endometriosis, even in the absence of symptoms that may suggest pelvic sites of the disease, in order to correctly map all possible implants of endometriosis according to the IDEA protocol and to appropriately manage the patient with medical and/or surgical treatment [34].

Regarding new ultrasonographic modalities, three-dimensional (3D) sonography has been proposed as a quick, non-invasive, reproducible, and cost-effective imaging modality to map extra-pelvic sites of endometriosis allowing for optimal surgical planning [35]. Through 3D US reconstruction the operator can characterize the lesion, reproducing its irregular shape and borders and also analyze the features of the surrounding tissue, the relationship between the nodule and the fascial plane and the infiltration of the fascia if present. A pre-operative estimate of the dimensions of the nodule, the volume, the depth of its localization and the infiltration of the abdominal wall structures is essential for the surgeon in order to perform an appropriate incision and to predict the need for a mesh prosthesis [35]. Obviously, in some cases MRI should be used in addition to evaluate the endometriotic lesion when ultrasonography is inconclusive or when the differential diagnosis appears to be particularly challenging and other pathologies need to be ruled out prior to surgery.

In conclusion ultrasonography seems to have a fundamental role in the majority of endometriosis in “atypical” sites in all the cases where “typical” clinical signs are present.
